# *Escherichia albertii* as a Potential Enteropathogen in the Light of Epidemiological and Genomic Studies

**DOI:** 10.3390/genes14071384

**Published:** 2023-06-30

**Authors:** Katarzyna Leszczyńska, Izabela Święcicka, Tamara Daniluk, Dariusz Lebensztejn, Sylwia Chmielewska-Deptuła, Dorota Leszczyńska, Jan Gawor, Małgorzata Kliber

**Affiliations:** 1 Department of Medical Microbiology and Nanobiomedical Enginnering, Medical University of Bialystok, ul. Mickiewicza 2C, 15-222 Bialystok, Poland; 2 Department of Microbiology and Biotechnology, University of Bialystok, ul. Ciołkowskiego 1J, 15-245 Białystok, Poland; 3 Department of Pediatrics, Gastroenterology, Hepatology, Nutrition, Allergology and Pulmonology, Medical University of Bialystok, ul. Waszyngtona 17, 15-274 Bialystok, Poland; 4 DNA Sequecing and Synthesis Facility, Institute of Biochemistry and Biophysics, Polish Academy of Sciences, ul. Pawińskiego 5A, 02-106 Warszawa, Poland; gaworj@ibb.waw.pl

**Keywords:** *Escherichia albertii*, enteropathogen, diarrhea, Shiga toxin, antibiotic susceptibility, intimin, pathogenicity island LEE, genome, pangenome

## Abstract

*Escherichia albertii* is a new enteropathogen of humans and animals. The aim of the study was to assess the prevalence and pathogenicity of *E. albertii* strains isolated in northeastern Poland using epidemiological and genomic studies. In 2015–2018, a total of 1154 fecal samples from children and adults, 497 bird droppings, 212 food samples, 92 water samples, and 500 lactose-negative *E. coli* strains were tested. A total of 42 *E. albertii* strains were isolated. The PCR method was suitable for their rapid identification. In total, 33.3% of *E. albertii* isolates were resistant to one antibiotic, and 16.7% to two. Isolates were sensitive to cefepime, imipenem, levofloxacin, gentamicin, trimethoprim/sulfamethoxazole, and did not produce ESBL β-lactamases. High genetic variability of *E. albertii* has been demonstrated. In the PFGE method, 90.5% of the strains had distinct pulsotypes. In MLST typing, 85.7% of strains were assigned distinct sequence types (STs), of which 64% were novel ST types. Cytolethal distending toxin (CDT) and Paa toxin genes were found in 100% of *E. albertii* isolates. Genes encoding toxins, IbeA, CdtB type 2, Tsh and Shiga (Stx2f), were found in 26.2%, 9.7%, 1.7%, and 0.4% of *E. albertii* isolates, respectively. The chromosome size of the tested strains ranged from 4,573,338 to 5,141,010 bp (average 4,784,003 bp), and at least one plasmid was present in all strains. The study contributes to a more accurate assessment of the genetic diversity of *E. albertii* and the potential threat it poses to public health.

## 1. Introduction

*Escherichia albertii (E. albertii)* is the second—after *Escherichia coli (E. coli)—*pathogenic species in the *Escherichia* genus, and is regarded as an emerging human and animal enteropathogen [1]. *E. albertii*, similarly to enteropathogenic *E. coli* (EPEC) and enterohemorrhagic *E. coli* (EHEC) strains, possesses a specific intestinal cell invasion mechanism known as A/E lesions (attaching and effacing lesions), characterized by intimate attachment of bacteria to the residual apical enterocyte membrane and localized destruction of brush border microvilli [2,3]. To date, only a limited number of publications are available about this bacterium due to late recognition of *E. albertii* as a new species (in 2003) and lack of standard identification methods [4]. Although this bacterium was isolated in the early 1990s and recognized as the cause of diarrhea in five Bangladeshi children, originally it was misidentified as *Hafnia alvei* [5]. However, further analyses of the isolates reveled substantial phenotypic and genomic differences in comparison to the reference *H. alvei* strains, and their relatedness to *Escherichia* and *Shigella*, particularly *Shigella boydii* B13. Moreover, the presence of the pathogenicity island LEE (locus of enterocyte effacement) carrying the *eae* gene that encodes intimin, a major virulence factor of EPEC/EHEC strains, was also noted in *E. albertii*. Further research gave rise to the creation of a new species in the Escherichia genus [6]. Nevertheless, *E. albertii* seems to be similar to pathogenic *E. coli* strains, not only in terms of virulence but also reservoirs and ways of transmission, where animals—particularly birds and contaminated food—possibly play an essential role [7,8]. Cytolethal distending toxin (CDT) and Shiga toxin (Stx) are the main types of toxins that have been reported in *E. albertii* strains. CDTs are genotoxins that induce DNA double-strand breaks in both proliferating and nonproliferating cells and are produced by several Gram-negative bacteria, such as *E. coli* and *Shigella* spp. [9]. CDT induces nuclear distension in infected cells and kills target cells. CDT consists of three subunits (CdtA, CdtB, and CdtC), with CdtB corresponding to the active subunit, while CdtA and CdtC form a heterodimeric subunit required for binding and intracellular delivery of CdtB to the target cells [10,11,12,13]. Some *E. albertii* strains possess the *stx* genes encoding Shiga toxins (Stxs). Stx inhibits protein synthesis in eukaryotic cells, and its production was originally identified in *Shigella dysenteriae* type 1 and later in Shiga toxin-producing *E. coli* strains [14]. Stxs are classified into two types, Stx1 (subtyped as 1a, 1c, and 1d) and Stx2 (subtyped as 2a, 2b, 2c, 2d, 2e, 2f, and 2g) [15]. All *stx*-positive *E. albertii* strains reported so far contain the *stx2f* gene [16,17,18], with the single exception of an *stx2a*-positive isolate in Norway [19]. Potential virulence factors also include Paa (porcine attaching and effacing-associated) toxin, IbeA toxin (brain microvascular endothelial cell invasion toxin), and Tsh toxin (temperature-sensitive hemagglutinin) responsible for the pathogenicity of avian pathogenic *E. coli* (APEC) [20]. The *paa* gene, which encodes the porcine attaching-effacing associated protein, is highly conserved in *E. albertii* strains [21]. The *ibe* genes are associated with invasion of brain endothelial cells. *ibeA* is described as unique to *E. coli* K1 strains [22]. In contrast, the epidemiology, transmission, prevalence and incidence of *E. albertii* infections remain unexplored [23]. The aim of the research was to determine the prevalence and spread of *E. albertii* and the resulting potential threat to public health for the Polish population. In addition, we aimed to determine the biochemical features and/or biomarkers useful in the rapid identification of *E. albertii*, as well as the assessment of drug susceptibility and the study of the pathogenic potential of *E. albertii* strains from northeastern Poland. We aimed to investigate the relationship between the origin of *E. albertii* strains and their virulence, i.e., the occurrence of individual pathotypes and/or commensal strains, as in the case of *E. coli*, and to determine whether the *E. albertii* pangenome is open, i.e., genome in which the number of gene families increases continuously as new genomes are added for analysis, similarly to the *E. coli* pangenome.

## 2. Materials and Methods

### 2.1. Collection of Samples and Bacterial Cultures

In 2015–2018, a total of 1154 fecal samples from children and adults, 212 food samples as well as 92 water samples were collected. The latter were gathered from various locations within the city of Bialystok’s water distribution system. In addition, 497 bird droppings, collected from otherwise healthy wild birds, i.e., without visible disease symptoms, in the area of (i) the Biebrza National Park, (ii) the Czeremcha commune, (iii) the Siemianowka Lagoon, and (iv) the city of Bialystok, were analyzed for the presence of *E. albertii*. The study material consisted of 580 stool samples collected from children and adults with acute and chronic diarrhea (as outpatients and/or hospital patients; the children were treated at the Department of Pediatrics, Gastroenterology, Hepatology, Nutrition, Allergology and Pulmonology of the Medical University of Bialystok) and 574 stool samples from children and adults without diarrhea (control group). The criterion for exclusion from the study was antibiotic therapy conducted 1 month before stool sample collection and lack of consent to participate in the study. The study was approved by the Bioethics Committee of the Medical University of Bialystok. In addition, we conducted a retrospective re-identification of 500 lactose-negative *E. coli* strains (a trait of *E. albertii*) from the collection of bacterial strains at the Department of Microbiology of the Medical University of Bialystok, isolated from the feces of patients (children and adults) hospitalized at the University Pediatric Hospital in Bialystok, the Municipal Hospital in Bialystok, and the Psychiatric Hospital in Choroszcz.

Human fecal samples (20–80 g) were collected in sterile containers, while sterile swabs with a transport medium were used to collect bird droppings. In the next step, all stool samples were plated on MacConkey, Hektoen, CHROMagar *E. coli* selective agar media (bioMerieux, France), blood agar, BHI broth (Oxoid, United Kingdom), and incubated at 37 °C for 48 h. In addition, fecal samples from human diarrhea were tested for common bacterial enteropathogens using culture methods on additional selective media: (i) SS and SF agar broth (bioMerieux, France) for *Salmonella* spp. and *Shigella* spp., (ii) selective *Campylobacter* agar (bioMérieux, France), and (iii) selective *Yersinia* agar (Merck, Germany). Commercial enzyme immunoassays or immunochromatography were used to identify *Clostridioides difficile*, norovirus and rotavirus/adenovirus infections. The purpose of these additional tests was to determine, in the case of *E. albertii* positive samples, whether this was the only etiological factor of the diarrhea.

Water samples (100 mL) were taken into sterile glass bottles and analyzed in accordance with the PN-EN ISO 9308-1:2004 procedure, based on the enumeration of *E. coli* and coliform bacteria in water by membrane filtration method and culture on selective Chromogenic Coliform Agar (CCA). Food samples were analyzed in accordance with PN-ISO 4832: 2007, i.e., the enumeration of *E. coli* and coliform bacteria using colony-count technique.

### 2.2. PCR Detection of E. albertii, Enteropathogenic E. coli (EPEC), Enterohemorrhagic E. coli (EHEC) and Avian Pathogenic E. coli (APEC) Strains

Single bacterial colonies isolated on agar media specific for enteric rods, i.e., MacConkey, Hektoen, CHROMagar *E. coli* selective agar, were subcultured on LB agar plates (Biomaxima, Lublin, Poland) and subjected to DNA isolation using Genomic Mini (A&A Biotechnology, Poland) followed by PCR screening for *E. albertii*, EPEC, EHEC and APEC strains. Firstly, in order to detect *E. albertii*, EPEC, and EHEC, primers for the *eae* gene were applied. Secondly, *E. albertii* specific primers, which recognize unique fragments of *lysP* and *mdh* genes, were used to differentiate this species from EPEC/EHEC strains [6]; thus, *eae*(+)*lysP*(+)*mdh*(+) strains were identified as *E. albertii*. On the other hand, primers for genes encoding Shiga toxins (*stx*) and *bfpA* gene were used to recognize (i) EHEC-*stx*(+), (ii) typical tEPEC-*stx*(−)*bfpA*(+), and (iii) atypical aEPEC-*stx*(−)*bfpA*(−), respectively. APEC strains were detected by multiplex PCR with primers for APEC-specific virulence genes, i.e., *astA*, *irp2, iss, papC, iucD* and *tsh* [24]. Reference strains *E. albertii* DSM 17582, EPEC ATCC 43887, DSM-8695 and *E. coli* O157:H7 NCTC 12900 served as positive controls. The amplification process was performed on the Mastercycler gradient (Eppendorf). All PCRs were performed in duplicate. Amplicons were visualized by electrophoresis on a 2% agarose gel stained with ethidium bromide, documented by GelDoc 2000 system (Bio-Rad), and identified based on the size comparison with amplicons from the reference strains and DNA markers.

### 2.3. Phenotypic Features of E. albertii

#### Biochemical Profiles

Biochemical features were determined based on API 50 CHE, API 20E and ID32E (bioMérieux, Marcy-l’Étoile, France) (also in order to determine the most common species identified by these commercial tests, which do not include *E. albertii*) using ATB Expression (bioMérieux). The tests were performed according to the manufacturer’s recommendations.

### 2.4. Antimicrobial Susceptibility Patterns

The susceptibility of each isolate was tested with disc diffusion against a panel of 10 antibiotic agents used in *E. coli* infections in human: ampicillin 10 µg/disc, amoxicillin-clavulanic acid 20–10 µg/disc, cefuroxime 30 µg/disc, cefepime 30 µg/disc, imipenem 10 µg/disc, chloramphenicol 30 µg/disc, ciprofloxacin 5 µg/disc, levofloxacin 5 µg/disc, gentamicin 10 µg/disc and trimethoprim/sulfamethoxazole 1.25–23.75 µg/disc (all antibiotics from Oxoid Ltd., Cambridge, UK) (Table 1). Susceptibility testing was conducted using clinical breakpoints on Mueller–Hinton agar according to European Committee on Antimicrobial Susceptibility Testing (EUCAST) recommendations, including the *E. coli* ATCC 25922 as a reference strain [25]. When resistance of the strains was detected, the MIC values were determined using E-tests (bioMérieux). The production of *E. albertii* extended-spectrum β-lactamases (ESBLs) was determined using the double-disk synergy test (DDST) [26]. Specifically, this was performed with cefotaxime (30 μg) and ceftazidime (30 μg) disks placed at a distance of 20 mm (center to center) from the amoxicillin-clavulanic acid disk (20/10 μg). A cefepime (30 μg) disk was placed in the same culture medium in order to improve the detection of ESBL during the simultaneous stable hyperproduction of an AmpC β-lactamase. The test result was considered positive when an enhancement of the inhibition zone around at least one of the antibiotic disks (cefotaxime, ceftazidime, or cefepime) toward the clavulanic acid disk was observed. The control strains *Klebsiella pneumoniae* ATCC 700603 (ESBL positive) and *E. coli* ATCC 25922 (ESBL negative) were used for quality control.

Determination of Genetic Relatedness

### 2.5. Serotyping

All *E. albertii* and *E. coli eae*-positive or APEC isolates were serotyped using standard *E. coli* O antisera (Biomed, Kraków, Poland).

### 2.6. Restriction Fragment Length Polymorphism (RFLP)

All *E. albertii* isolates were typed by pulse field gel electrophoresis (PFGE) with XbaI endonuclease according to the PulseNet protocol (http://www.cdc.gov/pulsenet/pdf/ecoli-shigella-salmonella-pfge-protocol-508c.pdf) using CHEF Mapper (Bio-Rad, Cressier, Switzerland). PFGE gels after staining with ethidium bromide were captured in the GelDoc 2000 system (Bio-Rad, Hercules, CA, USA) and analyzed with BioNumerics software ver. 7 (Applied Maths). PFGE patterns were compared using the Dice coefficient and unweighted pair group method using arithmetic averages (UPGMA) clustering, with a 1% band position tolerance window and 1% optimization. Isolates were defined as having a clonal relationship if they possessed 85% similarity to the PFGE patterns. Further analyses (except for plasmid profiling) were performed only for nonclonal strains.

### 2.7. Plasmid Profiles

Plasmid profiling assays are necessary from the perspective of virulence determination, strain typing and assembly of whole genome sequences. Therefore, the number and the size of *E. albertii* plasmids < 100 kb were determined using a commercial plasmid isolation kit (e.g., Qiagen Plasmid Plus Midi Kit; Qiagen) and regular gel electrophoresis. On the other hand, larger plasmids (>100 kb) were detected by means of the PFGE method after plasmid linearization by nuclease S1 [27].

### 2.8. Multilocus Sequence Typing (MLST)

MLST was performed for nonclonal *E. albertii* isolates based on seven housekeeping genes (*adk, fumC, gyrB, icd, mdh, purA, recA*) according to the protocol available at http://mlst.warwick.ac.uk/mlst/dbs/Ecoli/ (accessed on 27 April 2023) (Table 2).

### 2.9. Virulence Gene Sequencing

The genes common for *E. albertii* strains, e.g., *eae*, *cdtA-B*, *ibeA*, *tsh*, *vat*, *stx*, and *aida,* were sequenced using Expand long-template PCR kit (Roche, Switzerland) and ABI3500 genetic analyzer (Applied Biosystems). In addition, the expression level of major *E. albertii* virulence genes, notably associated with adhesion process and localized either on the pathogenicity island LEE, e.g., *eac*, *espA*, *ler*, or outside of it, e.g., *aida*, *paa*, were assessed using quantitative reverse transcriptase real-time PCR (qRT-PCR) and PikoReal termocycler (Termo Scientific); *rpoA* (RNA polymerase), *rrsG* (16S ribosomal RNA), and *arcA* (aerobic respiration control) served as reference genes.

### 2.10. Whole Genome Sequencing of E. Albertii Using Next-Generation Sequencing (NGS)

In total, genomes of 25 nonclonal *E. albertii* isolates were sequenced using short- and long-read sequencing with MiSeq (Illumina, San Diego, CA, USA) and MinION (Oxford Nanopore Technologies, Oxford, UK) instruments, respectively. In detail, the total genomic DNA of bacteria was extracted using the CTAB/lysozyme method [28], and for the short-read sequencing, Paired-End TruSeq-like libraries were constructed using KAPA Library preparation kit (KAPA/Roche, Basel, Switzerland) according to the manufacturer’s instructions, followed by sequencing in paired-end mode with MiSeq Reagent Kit v3 (600-cycle). MinION sequencing was performed using SQK-LSK108 chemistry and R9.4/R9.5 ONT flowcells. Raw nanopore data were basecalled using Albacore v2.0.2 (Oxford Nanopore Technologies, Oxford, UK). The obtained short and long reads were filtered by quality using FastX toolkit ver. 0.0.14 (http://hannonlab.cshl.edu/fastx_toolkit/) and Porechop ver. 0.2.4 (https://github.com/rrwick/Porechop/), respectively. Finally, de novo assembly of the genomes was performed using Canu ver. 1.6 [29], and the Canu assembled contigs were further polished using Illumina sequencing reads and Oxford Nanopore ont-assembly-polish pipeline (https://github.com/nanoporetech/ont-assembly-polish/; accessed on 21 April 2021). The remaining gaps in the genome assemblies were closed by the PCR amplification of DNA fragments, followed by Sanger sequencing with an ABI3730xl Genetic Analyzer (Life Technologies, Carlsbad, CA, USA) using BigDye Terminator Mix v. 3.1 chemistry (Life Technologies). All of the sequence errors and missassemblies were further corrected using Seqman software ver. 7 (DNAStar, Madison, WI, USA) to obtain complete nucleotide sequences of bacterial genomes. The complete sequences of chromosomes and plasmids have been deposited in the GenBank database (BioProject ID: PRJNA931640).

## 3. Data Analysis

### 3.1. Bioinformatic Analysis and Study of E. albertii Genomes

#### 3.1.1. Genome Annotation

Genome annotations (genes, tRNA, rRNA operons, mobile genetic elements, etc.) were performed using Prokka [30] and were manually corrected based on BLAST comparison with sequences available in the GenBank, and additional bioinformatic tools and databases, such as: IS Finder (insertion sequences identification; http://www-is.biotoul.fr/; accessed on 12 January 2021), ARDB (Antibiotic Resistance Genes Database; http: ardb.cbcb.umd.edu/; accessed on 12 January 2021) and VFDB (Virulence Factors of Bacterial Pathogens; http://www.mgc.ac.cn/VFs/; accessed on 12 January 2021), and ABRicate ver. 0.8 (https://github.com/tseemann/abricate).

#### 3.1.2. Pangenome Estimation

Roary software [31] was used to estimate the size of *E. albertii* pangenome and identify the core genes.

### 3.2. Statistical Analysis

The results from biochemical tests, PFGE, MLST, antimicrobial susceptibility patterns, and other binary or categorical variables were analyzed using statistical methods, e.g., cluster analysis with BioNumerics ver. 7 (Applied Maths), Statistica (StatSoft, Tulsa, OK, USA) and/or Stata (StataCorp, College Station, TX, USA) software.

## 4. Results

From the tested materials—(i) 497 samples of bird droppings, (ii) 1154 fecal samples from children and adults, (iii) 212 food samples, (iv) 92 samples of water from natural reservoirs and tap water—a total of 42 strains of *E. albertii* were isolated. *E. albertii* could not be isolated from the collected human stool samples, indicating that its distribution is mainly restricted to birds and the environment. 

**Figure 1:**
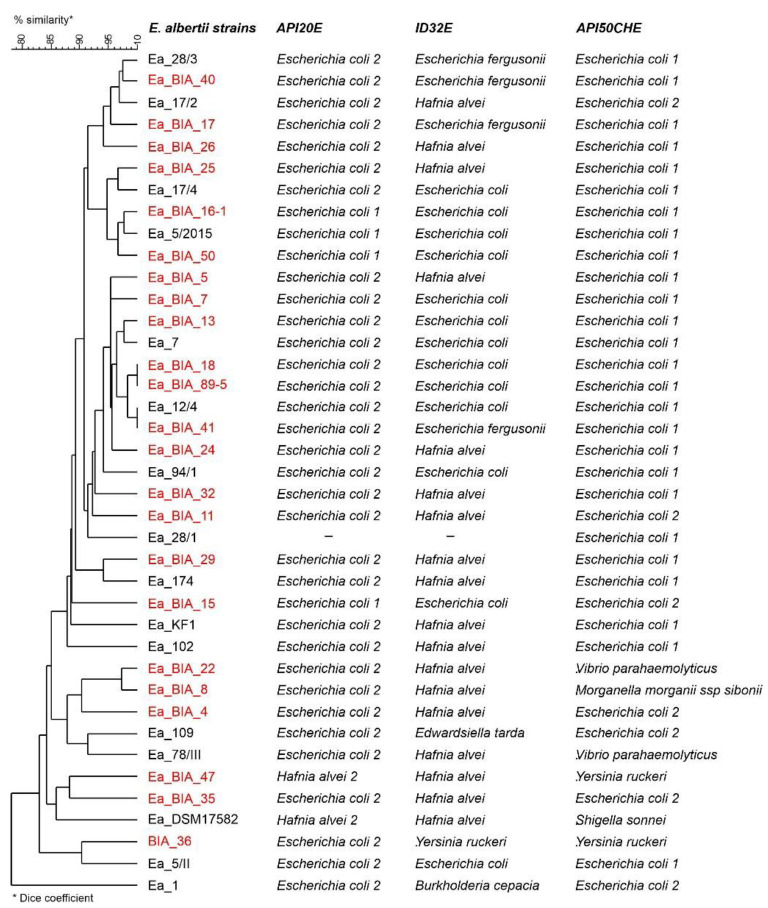
Similarity of *E. albertii* strains based on comparison of biochemical profiles determined based on API 50 CHE, API 20E and ID32E tests (bioMérieux). Dendrogram based on the average connection method (UPGMA) and the Dice similarity measure. *E. albertii* strains selected for WGS are highlighted in red color; Ea—*E. albertii.* Supplementary Table S1 (Biochemical profiles of *E. albertii* strains estimated based on API20E, ID32E and API50CHE tests).

In the present study, diagnostic PCR proved useful for rapid identification of the *E. albertii* strains, since different biochemical profiles were found in 38.1% of *E. albertii* isolates, which influenced their identification (Figure 1). Briefly, the studied strains of *E. albertii* were identified mainly as *Escherichia coli* type 1 or 2 and *Hafnia alvei*, and occasionally as *Escherichia fergusonii*, *Edwardsiella trada*, *Yersinia ruckei*, *Burkholderia cepacia*, *Vibrio parahaemolyticus*, and *Morganella morganii*. Unlike many *E. coli* strains, *E. albertii* strains failed to ferment a number of sugars, including lactose, dulcitol, L-rhamnose, and melibiose. The ability to ferment D-sorbitol, a trait almost exclusively associated with *E. coli* among *Escherichia* species, clearly helps separate this group (D-sorbitol negative) from the type species of the genus.

They can be biochemically distinguished from other *Escherichia* species by a number of tests, including the inability to utilize malonate and the lack of acid production from D-xylose, D-arabitol, melibiose, and cellobiose. We also found that the weak to moderate L-prolinoaminopeptidase activity noted in *E. albertii* strains may be a useful auxiliary test to separate this group from *H. alvei*. Because the species *E. albertii* was recently discovered, commercial systems do not currently include the species in their database or do not take into account the differences in their biochemical profiles. The results of testing 42 strains of *E. albertii* on various commercial systems suggest that a majority (80%) of the obtained biochemical profiles are matched with other species included in their databases with an unacceptable probability (around 60%) or are classified as unidentified. When identifying bacteria, the possible presence of *E. albertii* may be indicated by a result identifying the isolate as *H. alvei*, which is both L-rhamnose negative and D-xylose negative. Such results should trigger a more in-depth analysis of the test strain with additional testing. However, 45% of *E. albertii* isolates generated acceptable (92%) to perfect (97 to 99%) identifications as *H. alvei* or *E. coli.* Therefore, *E. albertii* strains may not be identified in routine diagnostics. 

When assessing drug susceptibility, we showed that, in total, 33.3% of the 42 tested *E. albertii* isolates were resistant to one antibiotic, and 16.7% of isolates showed resistance to two antibiotics. Most often, *E. albertii* was resistant to ampicillin (45.0%), followed by amoxicillin with clavulanic acid (9.0%), and very uncommonly to chloramphenicol (2.0%), ciprofloxacin and cefuroxime (5.0% each). However, none of the isolates were resistant to cefepime, imipenem, levofloxacin, gentamicin, or trimethoprim/sulfamethoxazole (Table 1). The tested *E. albertii* isolates did not produce extended-spectrum β-lactamases.

**Figure 2:**
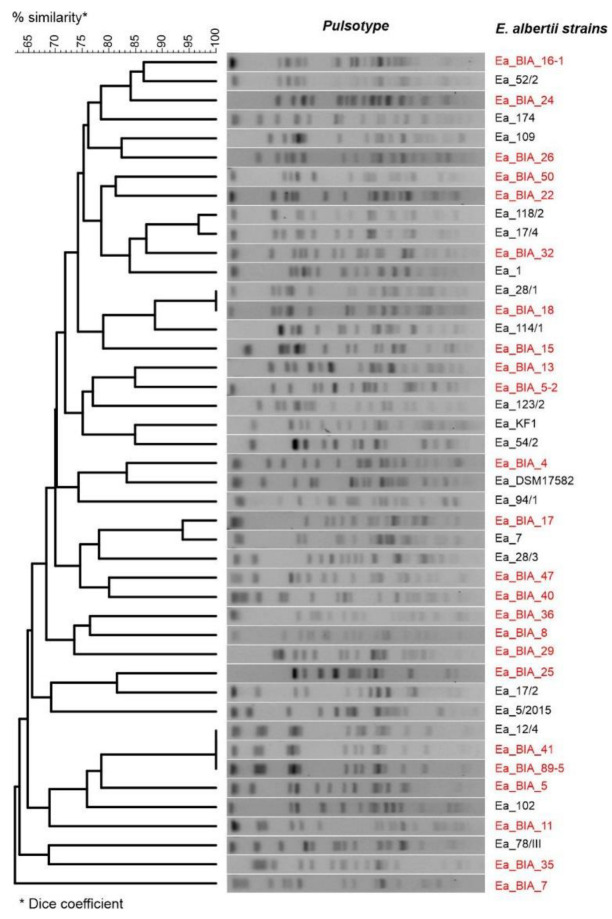
Genetic profiles (pulsotypes) of *E. albertii* (Ea) strains. Dendrogram based on the average connection method (UPGMA) and the Dice similarity measure. *E. albertii* strains selected for WGS are highlighted in red color; Ea—*E. albertii*.

Of the 42 isolated strains of *E. albertii*, we were able to obtain complete sequences of the genomes (chromosomes and plasmids) of 25 by sequencing using the new generation NGS. The characteristics of the sequenced strains are presented in Table 2. Similarly, genetic typing showed high variability in isolates. In the PFGE method, 90.5% of the strains had distinct pulsotypes (Figure 2). In contrast, in MLST typing, 85.7% of the strains were assigned distinct sequence types (STs), of which 64% were new ST types (Table 2).

**Figure 3:**
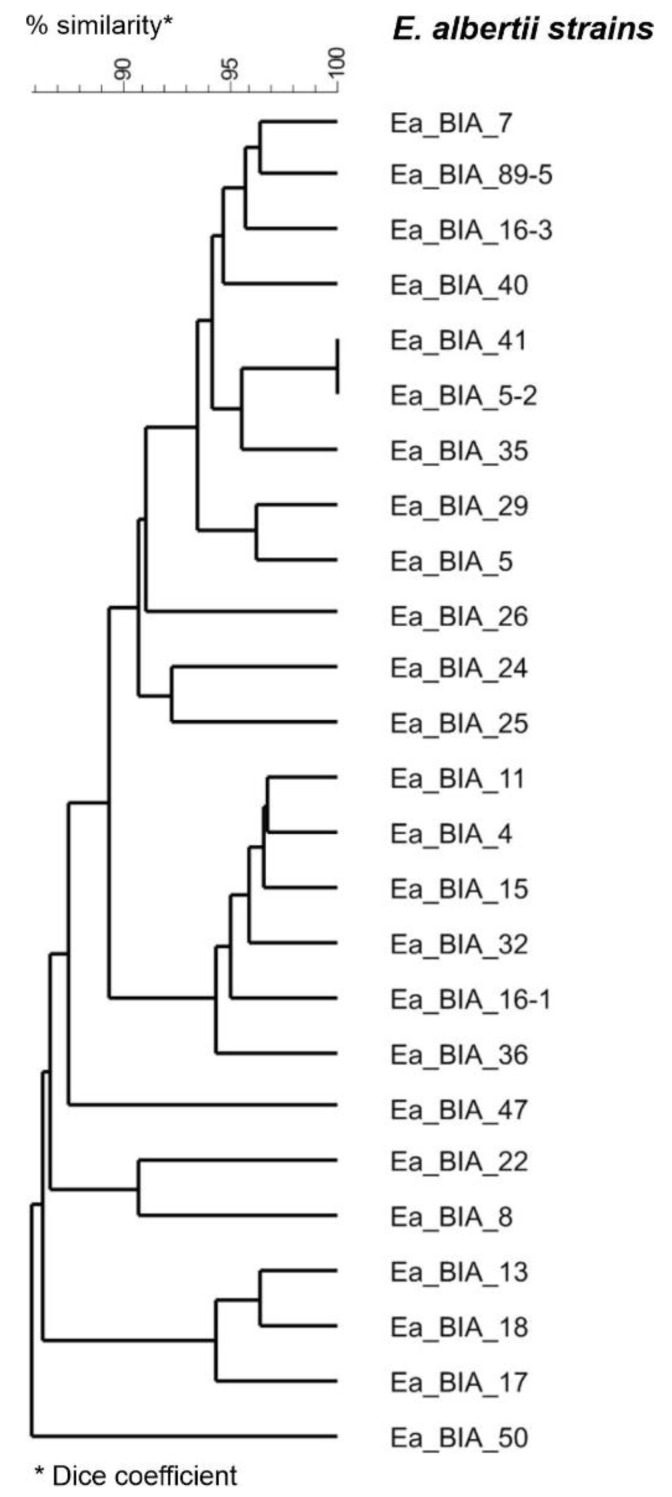
Similarity of sequenced strains of *E. albertii* (Ea) based on the presence of virulence genes (*E. coli*_vf n = 2710 and vfdb n = 3688 gene databases). Dendrogram based on the average connection method (UPGMA) and the Dice similarity measure. Analysis made in program ABRicate ver. 0.8 (https://github.com/tseemann/abricate).

In the case of typing virulence factors, the genes encoding cytolethal distending toxin (CDT) and porcine attaching and effacing-associated (Paa) toxins were demonstrated in 100% of the tested *E. albertii* isolates. On the other hand, the presence of genes encoding IbeA toxin (brain microvascular endothelial cell invasion toxin), CdtB type 2 toxin, Tsh toxin (temperature-sensitive hemagglutinin), and Shiga toxin (Stx2f) was found in 26.2%, 9.7%, 1.7% and 0.4% *of E. albertii* isolates, respectively. A comparison of the virulence profiles of the sequenced *E. albertii* strains based on bioinformatic database analysis virulence genes (*E. coli*_vf n = 2710 and vfdb n = 3688) is shown in Figure 3.

However, among the resistance genes, only those encoding β-lactamases were detected: blaEC-5 (4.8% of the strains), blaEC-8 (54.8% of the strains), and blaTEM-116 (7.1% of strains).

The chromosome size of the tested strains ranged from 4,573,338 to 5,141,010 bp (average 4,784,003 bp). In each strain, the presence of at least one plasmid (range 1–8, average 3.8) was found in the range 1589–177,310 bp (average 58,791.6 bp). Among the plasmids, 20 different types were detected replicons, which were dominated by IncF, IncI, IncX, IncY (46.9%), and Col (37.5%) replicons. The genome characteristics are presented in Table 3.

**Figure 4:**
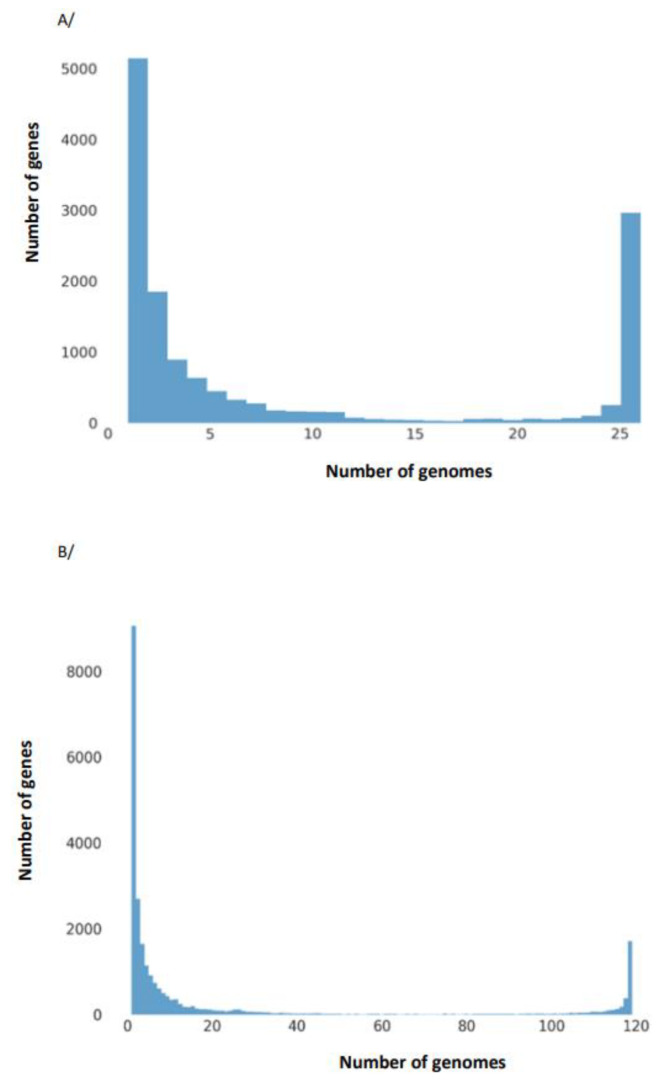
Graph showing the frequency of occurrence of genes included in the pangenome of *E. albertii* depending on the number of genomes. Analysis based on 26 (**A**) and 119 genomes (**B**) performed in Roary ver. 3.13.0.

**Figure 5:**
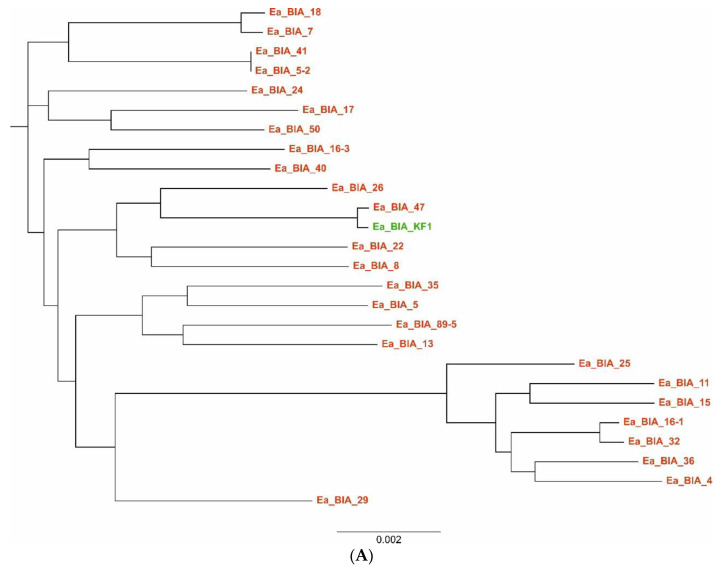

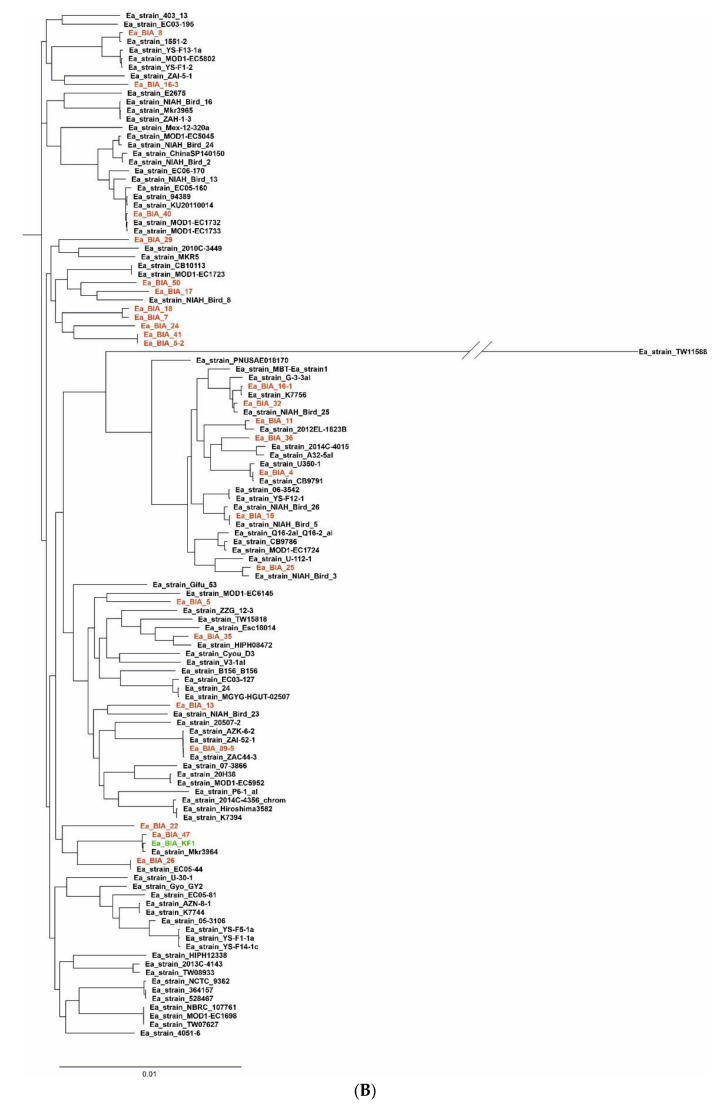
Phylogenetic similarity of *E. albertii* strains based on the analysis of genes included in the pangenome of 26 (**A**) and 119 strains (**B**). Twenty-five strains sequenced in the OPUS-9 project are marked in red, an earlier *E. albertii* isolate from our center (DOI: 10.1128/genomeA.00004-14) in green, strains from the GenBank database in black. Analysis made in Roary and FastTree programs. Ea—*E. albertii*.

The results of the pangenome analysis of the studied *E. albertii* isolates, taking into account the earlier isolate of *E. albertii* z of our center (DOI: 10.1128/genomeA.00004-14) and the 94 strains of *E. albertii* available in GenBank database (119 strains in total), are presented in Table 4 and Figure 4. Their phylogenetic relationship is shown in Figure 5.

## 5. Discussion

According to WHO estimates, diarrheal diseases are responsible for two billion deaths worldwide each year. In addition, infectious diarrhea is the leading cause of death among children under age 5 in developing countries [32], and in developed countries it is mainly the problem of morbidity and socio-economic costs [33]. Infections caused by enteropathogens can also result in serious complications, such as neurological or autoimmune diseases (Guillain–Barré syndrome). Unfortunately, a number of factors (demographic changes, centralization of food production, changes in eating habits, globalization, etc.), combined with the rapid adaptation of bacteria to new conditions, result in the formation of new enteropathogens [34].

*E. albertii* is considered a novel enteropathogen for humans and animals. Reports of the isolation of *E. albertii* from human and animals, the environment and food samples come from different geographical areas of the world, confirming its globalization. The clinical significance of *E. albertii* is not yet fully understood, partially because it is difficult to distinguish it from other *Enterobacteriaceae* using routine identification protocols. *E. albertii* was first isolated from infants in Bangladesh, and in subsequent years its involvement in infections in children and adults was confirmed [4,5,35]. In 2010, we also isolated *E. albertii* from the diarrheal feces of a child, but to date this has been a single case [23].

People infected with *E. albertii* usually show symptoms related to gastroenteritis, including watery diarrhea, dehydration, abdominal distension, vomiting, and in some cases fever. In most cases, these infections are self-limiting, with patients often recovering with little to no treatment [5,33,35]. However, a small subset of *E. albertii* isolates carry the *stx2a* allele and can cause bloody diarrhea [19]. Incubation periods of *E. albertii* infections that present as diarrhea are relatively short (average 12–24 h), while mortality rates are unknown. However, morbidity rates appear to be relatively high, i.e., >50% of the exposed population [36,37].

Other researchers, Afshin et al. [38] and Zaki et al. [39], also described cases of urinary tract infections with *E. albertii* as the etiological agent. In addition, a single case of *E. albertii* bacteremia was reported in an elderly woman with multiple comorbidities, possibly due to its ability to move from the intestinal lumen to other extraintestinal sites [35,40]. It may seem that these are relatively rare cases of *E. albertii* involvement in human infections; however, this may be due to misidentification.

The problem of increasing antimicrobial resistance among many bacteria should also be taken into account. *E. albertii* strains resistant to important antibiotics, including ampicillin, gentamicin, ciprofloxacin, norfloxacin, trimethoprim/sulfamethoxazole, rifampicin, meropenem and imipenem, were found [21,41,42,43]. In addition, multidrug-resistant strains of *E. albertii* [42] that showed resistance or coded for resistance to antibiotics from at least eleven classes [44,45] have been described. Combined with the virulence potential of this pathogen, these strains pose a serious threat to public health.

The data obtained from the conducted studies suggest that *E. albertii* may occur in animals or humans as a commensal or pathogen. *E. albertii* has been recognized as the etiological agent of diseases in many bird species [7]. In 1994, numerous dead finches were recorded in Scotland [46,47]. The necropsy showed that the causative agent was *E. coli* belonging to the O86:K61 serotype [46,47], which was previously associated with diarrhea in calves, pigs and horses and with cellulitis in broiler chickens [47]. However, a later study that re-examined these isolates with MLST and a larger panel of phenotypic and biochemical tests showed that it was in fact the *E. albertii* species [1,7]. In Australia, studies of vertebrate gut bacteria have also shown that birds (chickens and magpies) were the main reservoir of *E. albertii* [1,48,49]. Our research conducted in northeastern Poland also confirmed the relationship between the occurrence of *E. albertii* and migratory birds and the water bodies at which they stop. We isolated our strains mainly from the feces of cranes, starlings and geese, and from the water near their habitats. Pigeons were carriers of *E. albertii* in the urban environment. Our research confirmed the results of other researchers [50,51,52,53] that *E. albertii* is present in the wild bird population, which may be the potential source of this pathogen for humans, other animals, and the environment.

## 6. Conclusions

The isolates tested in our center do not currently constitute an important reservoir of resistance genes. We also found them to be highly sensitive to the most commonly used antibacterial drugs. However, in terms of virulence genes, the pathogenic potential of the studied *E. albertii* strains may range from watery diarrhea typical of enteropathogenic *E. coli* strains to bloody diarrhea typical of EHEC strains, due to the presence of genes encoding Shiga toxin. An issue requiring further research is the potential of *E. albertii* strains containing the IbeA toxin (characteristic of NMEC strains; *E. coli* neonatal meningitides) to cause infections of the central nervous system. In addition, their pathogenicity to animals—pigs—is also noteworthy due to the presence of the Paa toxin typical of porcine enteropathogenic *Escherichia coli (PEPEC*) strains. Further understanding of the pathobiology of *E. albertii,* as well as the mechanisms of colonization, survival, and dissemination within and between hosts, is still limited and requires further investigation. This will only be possible if simple and effective diagnostic tools are developed to enable its effective isolation and identification.

The study provides several important data regarding the epidemiology of *E. albertii*; for instance, we noted a high genetic similarity between our human isolate—Ea_BIA_KF1 [22] and strains isolated from the environment (Ea_BIA_47) and wild birds (Ea_BIA_22) as well as those described in other countries, e.g., Japan [36] (Figure 5). As a result, it helps for a better estimation of a risk that *E. albertii* may pose for public health. In the conducted studies, we confirmed the high genetic diversity of *E. albertii* strains, as is the case with *E. coli* strains, and the open nature of the pangenome of this bacterium, in which plasmids are the main mobile genetic factors responsible for its plasticity. They may play an important role in the horizontal exchange of virulence genes with *E. coli* strains pathogenic to animals and humans.

## Figures and Tables

**Table 1:** Drug susceptibility profiles of *E. albertii* strains isolated in northeastern Poland.

*E. albertii* Strains	Antibiotics									
	AM	AMC	CXM	CEF	IP	CH	CIP	LEV	GN	SXT
Ea_28/3; Ea_109; Ea_102; Ea_54/2; Ea_29/2; Ea_BIA_8; Ea_BIA_29; Ea_BIA_40; Ea_BIA_22; Ea_BIA_18; Ea_BIA_16-3; Ea_BIA_11; Ea_BIA_16-1;	**R**	**S**	**I**	**S**	**S**	**S**	**S**	**S**	**S**	**S**
Ea_174; Ea_28/1; Ea_78/III; Ea_7; Ea_15/2; Ea_17/4; Ea_5/2015; Ea_17/2; Ea_1; Ea_69/2; Ea_BIA_29; Ea_BIA_36; Ea_BIA_47; Ea_BIA_5; Ea_BIA_50; Ea_BIA_5-2; Ea_BIA_7; Ea_BIA_13; Ea_BIA_15; Ea_BIA_17; Ea_BIA_24;	**S**	**S**	**I**	**S**	**S**	**S**	**S**	**S**	**S**	**S**
Ea_12/4; Ea_94/2; Ea_BIA_89-5; Ea_BIA_26;	**R**	**R**	**I**	**S**	**S**	**S**	**S**	**S**	**S**	**S**
Ea_BIA_24	**R**	**S**	**I**	**S**	**S**	**R**	**S**	**S**	**S**	**S**
Ea_BIA_4	**R**	**S**	**R**	**S**	**S**	**S**	**S**	**S**	**S**	**S**
Ea_BIA_35	**S**	**S**	**R**	**S**	**S**	**S**	**R**	**S**	**S**	**S**
Ea_BIA_40	**S**	**S**	**I**	**S**	**S**	**S**	**R**	**S**	**S**	**S**

Abbreviations: AM, Ampicillin; AMC, Amoxicillin/Clavulanic acid; CXM, Cefuroxime; CEF, Cefepime; CH, Chloramphenicol; CIP, Ciprofloxacin; IP, Imipenem; GN, Gentamicin; LEV, Levofloxacin SXT, Trimethoprim/Sulfamethoxazole. I, susceptible—increased exposure; S, susceptible—standard dosing regimen; R, resistant strain. *E. albertii* strains selected for WGS are highlighted in red color; Ea—*E. albertii.*

**Table 2:** Characterization of sequenced *E. albertii* strains.

*E. albertii* Strains	Origin	Serotype	Type/Sequential Profile ^a^							
			ST	adk	fumC	gyrB	icd	mdh	purA	recA
Ea_BIA_11	Bird (*Sturnus vulgaris*)	H33:O119	11608	228	293	85	207	116	119	106
Ea_BIA_13	Bird (*Grus grus*)	H52:O28	nearest ST: 6059	163	395	134	386	378	444	~373 ^b^
Ea_BIA_15	Bird (*Passer domesticus*)	H52:O128	4736	228	170	85	332	116	149	106
Ea_BIA_16-1	Bird (*Sturnus vulgaris*)	H52:O128	5967	132	170	85	207	116	206	106
Ea_BIA_16-3	Bird (*Sturnus vulgaris*)	H5:-	nearest ST: 11622	228	788	85	~468 ^b^	378	149	~103 ^b^
Ea_BIA_17	Bird (*Grus grus*)	H52:-	nearest ST: 10710	133	~399 ^b^	85	189	142	141	176
Ea_BIA_18	Bird (*Grus grus*)	H52:O115	nearest ST: 1846	133	298	235	255	~190 ^b^	66	176
Ea_BIA_22	Bird (*Grus grus*)	H52:-	nearest ST: 2683 ^c^	567	~394 ^b^	134	1787	255	113	226
Ea_BIA_24	Bird (*Grus grus*)	H52:O69	nearest ST: 6059 ^c^	228	~788 ^b^	~85 ^b^	330	109	~69 ^b^	176
Ea_BIA_25	Bird (*Grus grus*)	H52:O1	nearest ST: 1338	~132 ^b^	170	85	207	154	149	135
Ea_BIA_26	Bird (*Grus grus*)	H33:O182	6058	539	788	134	483	255	149	75
Ea_BIA_29	Bird (unidentified species)	H5:-	nearest ST: 11971	132	1587	134	~872 ^b^	256	66	76
Ea_BIA_32	Bird (*Delichon urbicum*)	H5:O8	nearest ST: 5399 ^c^	~379 ^b^	~170 ^b^	85	207	116	206	106
Ea_BIA_35	Bird (*Anser* spp.)	H52:O152	10709	163	1283	852	189	68	66	225
Ea_BIA_36	Bird (unidentified species)	H33:O115	11195	228	1645	85	332	116	119	106
Ea_BIA_4	Bird (unidentified species)	H33:O1	6049	537	170	85	332	116	206	106
Ea_BIA_40	Bird (*Anser* spp.)	H52:-	3762	132	394	134	144	110	66	102
Ea_BIA_41	Bird (*Sturnus vulgaris*)	H52:-	nearest ST: 12964	132	298	134	~189 ^b^	885	206	~226 ^b^
Ea_BIA_47	Backwaters (Jancewicze)	H33:-	7365	695	111	85	483	109	149	75
Ea_BIA_5	Bird (unidentified species)	H52:-	8691	163	393	85	189	68	246	125
Ea_BIA_50	Bird (*Grus grus*)	H52:O9	nearest ST: 10710 ^c^	~393 ^b^	104	~85 ^b^	~545 ^b^	111	141	~176 ^b^
Ea_BIA_5-2	Bird (*Columba livia*)	H33:O9	nearest ST: 12964	132	298	134	~189 ^b^	1238	206	885
Ea_BIA_7	Bird (*Columba livia*)	H52:O115	1846	163	298	235	255	190	66	176
Ea_BIA_8	Bird (*Columba livia*)	H52:O131	2681	134	164	85	169	254	69	103
Ea_BIA_89-5	Bird (*Grus grus*)	H52:O3	2680	293	395	85	330	68	248	226

^a^ according to the MLST profile for *E. coli*; analysis was performed with MLST 2.0 server (https://cge.food.dtu.dk/services/MLST/; accessed on 21 April 2021). ^b^ alleles with less than 100% identity. ^c^ more than one nearest ST matched.

**Table 3:** Genome characterization of sequenced *E. albertii* strains.

*E. albertii* Strains	Chromosome Size	Number of Genes	Number of Plasmids	Plasmid Size
Ea_BIA_11	4,779,775	4495	3	5952–110,945
Ea_BIA_13	4,819,152	4503	7	1589–128,726
Ea_BIA_15	4,772,054	4581	4	5140–130,064
Ea_BIA_16-1	4,573,338	4248	3	2733–99,447
Ea_BIA_16-3	4,838,644	4548	2	5055–82,983
Ea_BIA_17	4,854,152	4728	3	1590–132,593
Ea_BIA_18	4,871,752	4587	5	2760–144,030
Ea_BIA_22	4,884,955	4718	8	1590–152,064
Ea_BIA_24	4,841,726	4513	7	1589–136,544
Ea_BIA_25	4,810,099	4480	4	1589–100,741
Ea_BIA_26	4,654,873	4279	4	4703–114,713
Ea_BIA_29	4,687,526	4362	2	4593–116,006
Ea_BIA_32	4,822,417	4570	5	3758–125,136
Ea_BIA_35	4,725,775	4422	4	2571–88,900
Ea_BIA_36	4,790,901	4588	4	40,185–103,275
Ea_BIA_4	4,776,253	4548	4	36,239–97,069
Ea_BIA_40	4,826,552	4550	3	2719–95,160
Ea_BIA_41	4,677,218	4327	2	13,675–76,945
Ea_BIA_47	4,595,293	4288	2	2720–40,979
Ea_BIA_5	4,782,897	4330	4	3909–143,188
Ea_BIA_50	4,722,615	4365	4	1627–165,614
Ea_BIA_5-2	4,681,166	4356	1	76,945
Ea_BIA_7	4,960,105	4706	5	2283–177,310
Ea_BIA_8	4,709,836	4505	3	47,425–108,071
Ea_BIA_89-5	5,141,010	4990	1	75,610

**Table 4:** *E. albertii* pangenome estimated on the basis of genomic analysis of 26 tested strains (A) and an additional 94 from GenBank (B). The analysis was made in Roary ver. 3.13.0.

(A)		
Type Genes	No. Strains (N = 26 *)	No. Genes
Core genes	(99% ≤ strains ≤ 100%)	2966
Soft core genes	(95% ≤ strains < 99%)	252
Shell genes	(15% ≤ strains < 95%)	3062
Cloud genes	(0% ≤ strains < 15%)	7888
**Total**	**(0% ≤ strains ≤ 100%)**	**14,168**
* included previous *E. albertii* isolate from our center was used (DOI: 10.1128/genomeA.00004-14).		
**(B)**		
**Type Genes**	**No. Strains (N = 119)**	**No. Genes**
Core genes	(99% ≤ strains ≤ 100%)	2088
Soft core genes	(95% ≤ strains < 99%)	511
Shell genes	(15% ≤ strains < 95%)	3052
Cloud genes	(0% ≤ strains < 15%)	19,506
**Total**	**(0% ≤ strains ≤ 100%)**	**25,157**

## Data Availability

The first complete genome sequence of Escherichia albertii strain KF1, a new potential human enteric pathogen was published in Genom. Announcement 2014, 2, e00004–e00014. https://doi.org/10.1128/genomeA.00004-14.

## Supplementary Materials

The following supporting information can be downloaded at: https://www.mdpi.com/article/10.3390/genes14071384/s1, Table S1 (Biochemical profiles of *E. albertii* strains estimated based on API20E, ID32E and API50CHE tests).
